# An integrated solution of deep reinforcement learning for automatic IMRT treatment planning in non-small-cell lung cancer

**DOI:** 10.3389/fonc.2023.1124458

**Published:** 2023-02-03

**Authors:** Hanlin Wang, Xue Bai, Yajuan Wang, Yanfei Lu, Binbing Wang

**Affiliations:** Department of Radiation Physics, Zhejiang Key Laboratory of radiation Oncology, The Cancer Hospital of the University of Chinese Academy of Sciences (Zhejiang Cancer Hospital), Hangzhou, Zhejiang, China

**Keywords:** automatic treatment planning, deep reinforcement learning, integrated solution, multi-objectives adjustment policy network, intensity-modulated radiation therapy

## Abstract

**Purpose:**

To develop and evaluate an integrated solution for automatic intensity-modulated radiation therapy (IMRT) planning in non-small-cell lung cancer (NSCLC) cases.

**Methods:**

A novel algorithm named as multi-objectives adjustment policy network (MOAPN) was proposed and trained to learn how to adjust multiple optimization objectives in commercial Eclipse treatment planning system (TPS), based on the multi-agent deep reinforcement learning (DRL) scheme. Furthermore, a three-dimensional (3D) dose prediction module was developed to generate the patient-specific initial optimization objectives to reduce the overall exploration space during MOAPN training. 114 previously treated NSCLC cases suitable for stereotactic body radiotherapy (SBRT) were selected from the clinical database. 87 cases were used for the model training, and the remaining 27 cases for evaluating the feasibility and effectiveness of MOAPN in automatic treatment planning.

**Results:**

For all tested cases, the average number of adjustment steps was 21 ± 5.9 (mean ± 1 standard deviation). Compared with the MOAPN initial plans, the actual dose of chest wall, spinal cord, heart, lung (affected side), esophagus and bronchus in the MOAPN final plans reduced by 14.5%, 11.6%, 4.7%, 16.7%, 1.6% and 7.7%, respectively. The dose result of OARs in the MOAPN final plans was similar to those in the clinical plans. The complete automatic treatment plan for a new case was generated based on the integrated solution, with about 5-6 min.

**Conclusion:**

We successfully developed an integrated solution for automatic treatment planning. Using the 3D dose prediction module to obtain the patient-specific optimization objectives, MOAPN formed action-value policy can simultaneously adjust multiple objectives to obtain a high-quality plan in a shorter time. This integrated solution contributes to improving the efficiency of the overall planning workflow and reducing the variation of plan quality in different regions and treatment centers. Although improvement is warranted, this proof-of-concept study has demonstrated the feasibility of this integrated solution in automatic treatment planning based on the Eclipse TPS.

## 1 Introduction

A major advance in radiotherapy technology is the application of intensity modulated radiation therapy (IMRT) as one of the principal delivery techniques (1). Current IMRT plans are usually inversely planned using treatment planning systems (TPS) (2), to safely deliver uniform dose to the target, while minimizing damage to the nearby healthy tissues and organs at risk (OARs).

In the whole plan design process, plan optimization is integral to inverse treatment planning, which is a trial-and-error process to find a good set of optimization objectives, including weighting factors, dose limits, and volume constraints. Among them, the patient-specific optimization objectives are critically important for the creation of a high-quality plan. In the clinical workflow, the trial-and-error process is usually a manual, tedious and time-consuming task. In addition, the final plan quality is dependent on planners’ experience, planning difficulty and available time (3). Plans are often accepted under clinical pressure, although further improvement is still possible.

To address these issues, the concept of automatic planning has been proposed and implemented using a range of methodologies (4). Among the most commonly used approach is protocol based automatic iterative optimization (PB-AIO), which is designed to mimic the planning operations of the physicians by some artificial protocols (5). Earlier works on the iterative improvement of optimization objective/weights built on the seminal work of Xing et al (6). This approach is often combined with optimization algorithms in commercial TPSs (7) (8) (9), such as RayStation (RaySearch Laboratories AB, Stockholm, Sweden), Pinnacle (Philips Healthcare GmbH, Hamburg, Germany) and Eclipse (Varian Medical Systems, Palo Alto, CA). Another approach is knowledge-based planning (KBP), which directly utilizes prior knowledge and experience to either predict an achievable dose for a new patient from a similar population or to derive a better starting point for the further optimization. The commercial Eclipse TPS uses the KBP-based RapidPlan module in automatic planning for various tumor sites to estimate two-dimensional dose-volume histograms (DVH) (10) (11). In addition, deep learning prediction model is a new solution that uses neural networks to analyze spatial features and achieve patient-specific three-dimensional (3D) dose distribution (12). The dose prediction model has been applied to automate treatment planning in some studies (13) (14).

Recently, deep reinforcement learning (DRL) has showed exceptional performance in some sequential decision-making problems. Notably, it outperformed human experts in Atari games (15) and made a breakthrough in Go (16). In TPS, searching for the optimal optimization objectives of treatment planning through trail-and-error method is essentially a sequential decision-making problem. Compared with PB-AIO and KBP approaches, the DRL model is universal and does not rely on previous training experience. To date, the feasibility of DRL in treatment planning has been demonstrated in preliminary studies (17) (18). Pu et al. developed an intelligent DRL-based brachytherapy treatment planning framework that can utilize the learned dwell time adjustment policy to obtain a satisfactory plan (19). Shen et al. proposed the virtual treatment planner network based on an in-house TPS (20). By an end-to-end training, the network could operate in-house TPS parameters to generate high-quality plans. Previous studies showed that the DRL framework can support decisions for certain tasks in a human-like fashion to achieve similar or even better performance compared with humans.

Although the DRL-based solutions to the sequential decision-making problem of treatment planning are encouraging, the training efficiency of this model is a major concern. Clinically, the more adjustable optimization objectives need to be added when the complexity of treatment planning increases. Depending on a single-agent model, treatment planning selects one objective to adjust in each optimization. In this scenario, a longer time is required to solve the treatment planning optimization problems, which considerably prolongs the training process. In this paper, we proposed a multi-objectives adjustment policy network (MOAPN) based on a multi-agent DRL scheme, and further explored its feasibility to adjust multiple objectives in commercial Eclipse TPS. Furthermore, we developed a 3D dose prediction module, which worked with MOAPN to form an integrated solution for automatic treatment planning. The patient-specific initial optimization objectives were generated based on the predicted 3D dose distribution of each patient case. Therefore, the overall exploration space of each treatment planning could be reduced during MOAPN training. This study aimed to demonstrate the feasibility of this approach to automatic stereotactic body radiotherapy (SBRT) planning for lung cancer.

## 2 Material and methods

### 2.1 Patient cases collection

The 114 peripheral non-small-cell lung cancer (NSCLC) cases were retrospectively selected from the clinical database as the total study cohort, dated from June 2018 to March 2022. Two or more radiotherapists agreed that they were suitable for SBRT. Lung scanning was performed using a GE LightSpeed-RT simulator or a Philips large-aperture CT simulator. The clinical target volume (CTV), lung, chest wall, esophagus, bronchus, spinal cord and heart were delineated by experienced radiation oncologists. Due to set-up errors and organ motion, the planning target volume (PTV) was obtained by expanding 5 mm of the CTV in the 3D direction. The prescribed dose was 50 Gy spread over 5 fractions, and scaled to cover 95% of the PTV for all cases.

### 2.2 Auto-planning creation

In this study, the proposed automated optimization process of SBRT plans was implemented with Eclipse Scripting Application Programming interface (ESAPI) provided by the Eclipse TPS. Python-based scripts were developed in a research mode to implement the proposed automatic planning procedure for NSCLC patients, including plan creation, parameters setting & modification, plan optimization, data reading, and plan evaluation. All studied cases were preformed on a Varian True-Beam linear accelerator with a coplanar beam energy of 6 MV, equipped with the Millennium 120 MLC. All treatment plans were uniformly designed as IMRT and the angular interval of the fields was set to 40°.

In the inverse SBRT planning process, dose-limiting rings are often introduced (21) and used to control the dose gradient outside the target to an acceptable level. In this study, five 3D ring structures with a width of 4 mm were generated at distance of 0.3 cm, 1.0 cm, 2.4 cm, 4.4 cm and 6 cm outside the PTV, named Ring1, Ring2, Ring3, Ring4 and Ring5, respectively. These ring structures were used to participate in planning optimization to control the dose gradient outside the target and spare the normal tissues surrounding the target as much as possible. Moreover, an additional ring structure named D2cm was also generated by a prewritten script to evaluate the result of dose gradient. This ring structure with a width of 1 cm was obtained by expanding 2 cm outside the PTV in the 3D direction. All settings and requirements were the same between different studied cases. For the target, 100% prescription dose need to cover 95% of the PTV. For the OARs, the dose constraint followed the recommendations of radiation oncology working group (RTOG) 0915 report (22). The OAR dose was expected to be as low as possible without compromising the dose coverage to the target.

### 2.3 Optimization objective function

In this paper, the Eclipse Photon optimization (PO) algorithm (23) was used as the optimizer. The optimization was currently performed on an overall objective with multiple terms of dose volume histogram (DVH) constraints. Each DVH constraint designed for various considerations, corresponding to a set of planning optimization objectives, was composed of four input parameters: an optimization weight factor, a 2D-position on the DVH-graph representing the dose-volume objective, and a Boolean variable describing the direction of the constraint (upper of lower) on the DVH curve. If the dose-volume objectives are not met, a weighted quadratic cost is calculated, as follow:


(1) $$cost_{d}=\frac{1}{2}w\times {\left(D_{actual}-D_{obj}\right)}^{2}$$

Where *D_actual_* and *D_obj_* refer to the actual dose after optimization and the desired objective (e.g. *V*_20_, relative volume of the lung receiving doses of > 20 Gy should be less than 15%) before optimization, respectively. is a weight factor of the dose-volume objective. For the upper objective, the cost is applied for the portion of doses that exceed the desired dose value and volume level. For the lower objective, the cost is applied for the portion of doses that fall short of the desired dose value and volume level.

In addition, an alternative solution of the dose-volume objectives is the generalized form of equivalent uniform dose (gEUD) (24). Compared with physical dose, the gEUD considers radiobiologic factors and has the potential to improve the sparing of the critical structures in IMRT (25). However, the target is insensitive to the existence of hot spots within the gEUD optimization, and potentially has worse dose coverage. Then, optimization evaluates the gEUD values for the structure, and a square law cost is applied when an objective is not met in the same manner as in Eq. (1):


(2) $$cost_{gEUD}=w\times {\left(gEUD_{actual}-D_{EUD}\right)}^{2}$$

Where w is a weight factor of the gEUD objective.*D_EUD_* is the desired objective for EUD. The *gEUD_actual_* represents the actual value for EUD after optimization, as follows:


(3) $$\text{gEUD}={\left(\frac{1}{V}\times \sum_{V}D{\left(x\right)}^{a}\right)}^{\frac{1}{a}}$$

Where a is a biological parameter controlling the dose distribution inside the structure. Typical values range from -40 to 40. For serial structures (e.g., spinal cord, esophagus, chest wall) or ring structures, the gEUD parameter is assigned to a large positive value (a = 40) that tends to near the maximum dose. For parallel structures such as lung, the value of a would be small and positive (a = 1), the dose response may be more closely related to the mean dose (26). V is the volume of the structure. D(x) represents the dose in position x inside the volume V. For the lower gEUD objective, the cost value is 0 when gEUD > *D_EUD_*. For the upper gEUD objective, the cost value is 0 when gEUD > *D_EUD_*.

In Eclipse TPS, the optimization of an IMRT plan using PO optimizer was defined as a minimization problem. To obtain the ideal dose coverage and spare the critical structures, a hybrid objective function combined gEUD and physical dose constraints is developed as:


(4) $$\begin{array}{l} \underset{D}{\text{min}}(\omega_{L}^{PTV}{\left(D_{actual}^{PTV}-D_{p}\right)}_{lower}^{2}+\omega_{H}^{PTV}{\left(D_{actual}^{PTV}-D_{max}\right)}_{upper}^{2}+\\ \sum_{i}\omega_{i}^{OAR}{\left(gEUD_{i}^{OAR}-D_{EUD}^{i}\right)}_{upper}^{2}+\sum_{j}\omega_{j}^{ring}{\left(gEUD_{j}^{ring}-D_{EUD}^{j}\right)}_{upper}^{2}) \end{array}$$

Where $\omega_{L}^{PTV}$ and $\omega_{L}^{PTV}$ are weights for the two objectives of the PTV, respectively. *D_p_* and *D_max_* are the prescription dose and the maximum dose for the PTV. $\omega_{i}^{OAR}$ is a weight of the *i^th^* OAR, including chest wall, spinal cord, esophagus, bronchus, heart and lung, respectively. $\omega_{j}^{ring}$ is a weight of the *j^th^* ring structure, including Ring1, Ring2, Ring3, Ring4 and Ring5, respectively.

### 2.4 Dose prediction module

At the initial step of optimization, the clinical planners are accustomed to loading a set of predefined objectives generated by plan experience templates into the PO optimizer, performing the first optimization. These predefined objectives represent that a set of fixed optimization parameters is applied to a large number of patient cases, even with various variations between them. A set of good initial optimization objective parameters can not only achieve good optimization result, but also shorten the optimization process and improve work efficiency. In this study, a dose prediction module was developed to predict the 3D dose distribution and obtain the patient-specific initial optimization objective parameters.

With previous experiences on dose prediction (27), a dose prediction module based on U-Net architecture was configured with 76 clinical treated SBRT plans for peripheral NSCLC cases (60 cases for training and 16 cases for validation). All training plans were manually created by senior physicians following consistent dose prescription and planning protocols. The U-Net model had a total of 10 layers and all convolutional layers applied a 3×3×3 kernel except the output layer with 1×1×1 kernel. The input data was contours of the planning structures, which were converted from DICOM format files. It was a 3D matrix (64×256×256) with one channel, including PTV, lung, chest wall, esophagus, bronchus, spinal cord and heart, respectively. According to the structure type, each voxel was assigned a specific value (e.g. 1.0 for PTV, 0.88 for heart, 0.75 for spinal cord, 0.63 for esophagus, 0.5 for chest wall, 0.43 for bronchus and 0 for the voxel outside of the body). The output data was the 3D predicted dose distribution. The model was implemented in Keras, and the Adam optimization algorithm was used for the sharp loss function minimization (28).

**Figure 1:**
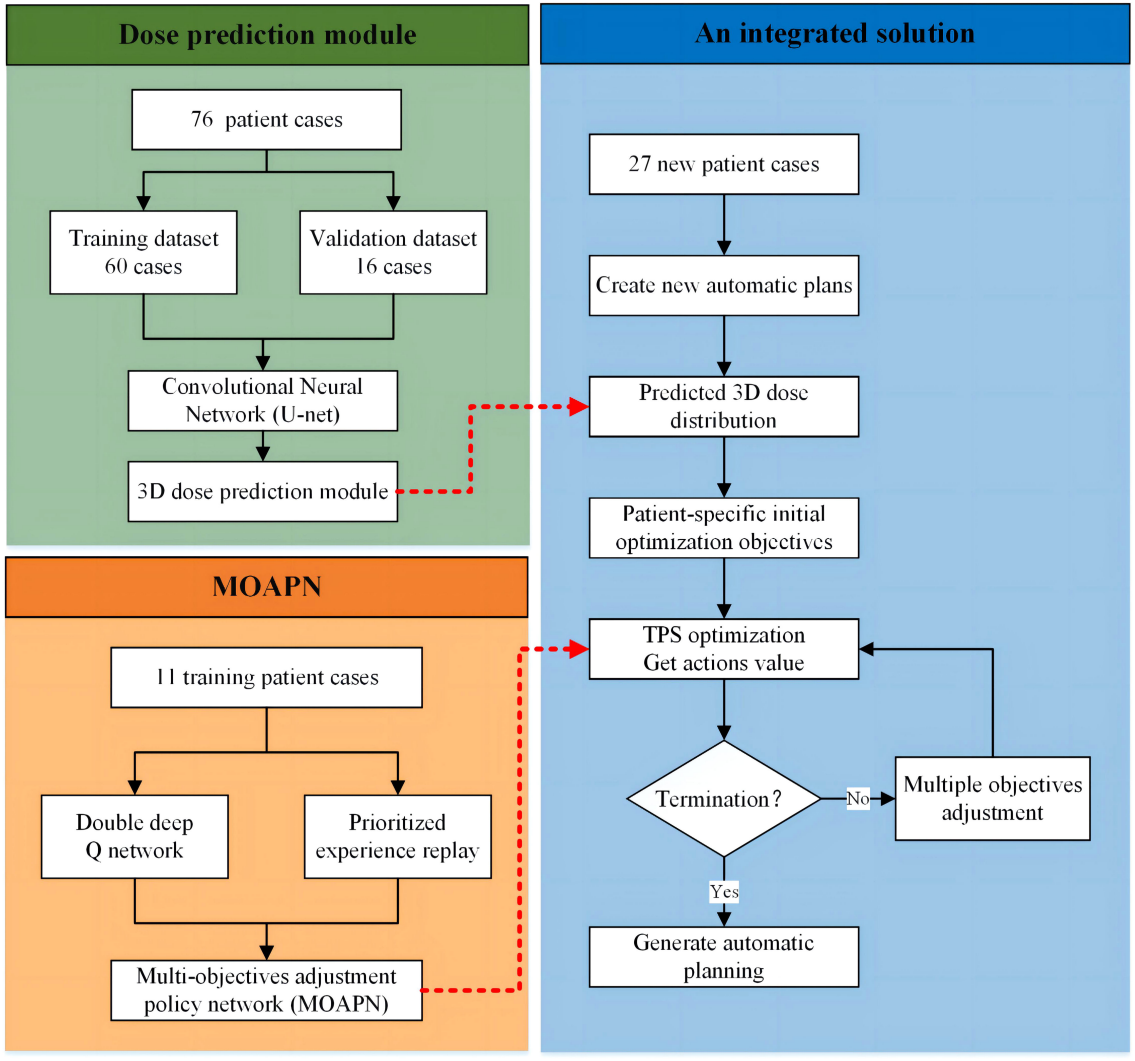
The overall workflow of an integrated solution for automatic treatment planning.

A complete dose prediction module is composed of the following three steps, as shown in **Figure 1**. First, using previously treated plans as training data generated DL model to predict the 3D dose distribution. Second, the new case’s CT with structure information of the target and OARs was used as inputs to automatically generate the 3D dose distribution. Third, based on the 3D dose distribution predicted by new cases, a prewritten script was utilized to count the dose values obtained for each dose voxel in the entire dose grid. The statistical results would form a 2D dose-volume histogram, yielding smooth dose-volume curves for all structures by interpolation. Referring to the clinical guidelines, the specific dose values or volume values for each structure (e.g. lung V_5_ and spinal cord D*_max_*) were selected in dose-volume curves and converted into initial optimization objective values, e.g. ${\text{D}}_{EUD}^{i}$ and ${\text{D}}_{EUD}^{j}$ , to participate in the optimization in Eq. (4).

### 2.5 Multi-objective adjustment policy network

#### 2.5.1 Network architecture

To build the proposed MOAPN, we used the Q-learning framework, which is a reinforcement learning algorithm (29) for solving the Markov Decision Processes (30). This framework tries to build the optimal action-value policy and its updated formula is:


(5) $$\text{Q}\left(s_{t},a_{t}\right)\leftarrow \text{Q}\left(s_{t},a_{t}\right)+\text{α}\left(r_{t}+\text{γmaxQ}\left(s_{t+1},a_{t+1}\right)-\text{Q}\left(s_{t},a_{t}\right)\right)$$

Where S*_t_* and S*_t_*_+1_ are the states at t and t+1 steps; a*_t_* and a*_t_*_+1_ are the actions at t and t+1 steps; r*_t_* represents reward value at t step; γ is the discount factor; α is the learning rate.

For large state spaces and action spaces, the standard Q-learning algorithm cannot bear huge computational burden. Thus, deep Q network (DQN) (15) is proposed to approximate action value function *via* a multi-layered neural network. Generally, the loss function of DQN is defined as:


(6) $$L_{i}\left(\theta_{i}\right)={\left[r+\gamma max_{a_{t}}Q\left(s_{t+1},a_{t+1};\theta_{i}^{tar}\right)-Q\left(s_{t},a_{t};\theta_{i}^{eva}\right)\right]}^{2}$$

Where $\theta_{i}^{eva}$ and $\theta_{i}^{tar}$ are the evaluation-network parameter and the target-network parameter, respectively.

To avoid overestimation of action values in the training process, the double-deep Q network (DDQN) (31) was proposed to decouple the selection of target Q-value actions and the calculation of target Q-value. The updated action-value function for DDQN algorithm is as follows:


(7) $$Q_{t}^{DDQN}=R_{t+1}+\gamma Q\left(s_{t+1},argmax_{a_{t}}Q\left(s_{t+1},a_{t};\theta_{i}^{eva}\right);\theta_{i}^{tar}\right)$$

In this study, we developed MOAPN based on the network architecture of DDQN. Additionally, the experience replay and fix Q target techniques were used in MOAPN to stabilize the training process.

#### 2.5.2 States and actions

**Figure 2:**
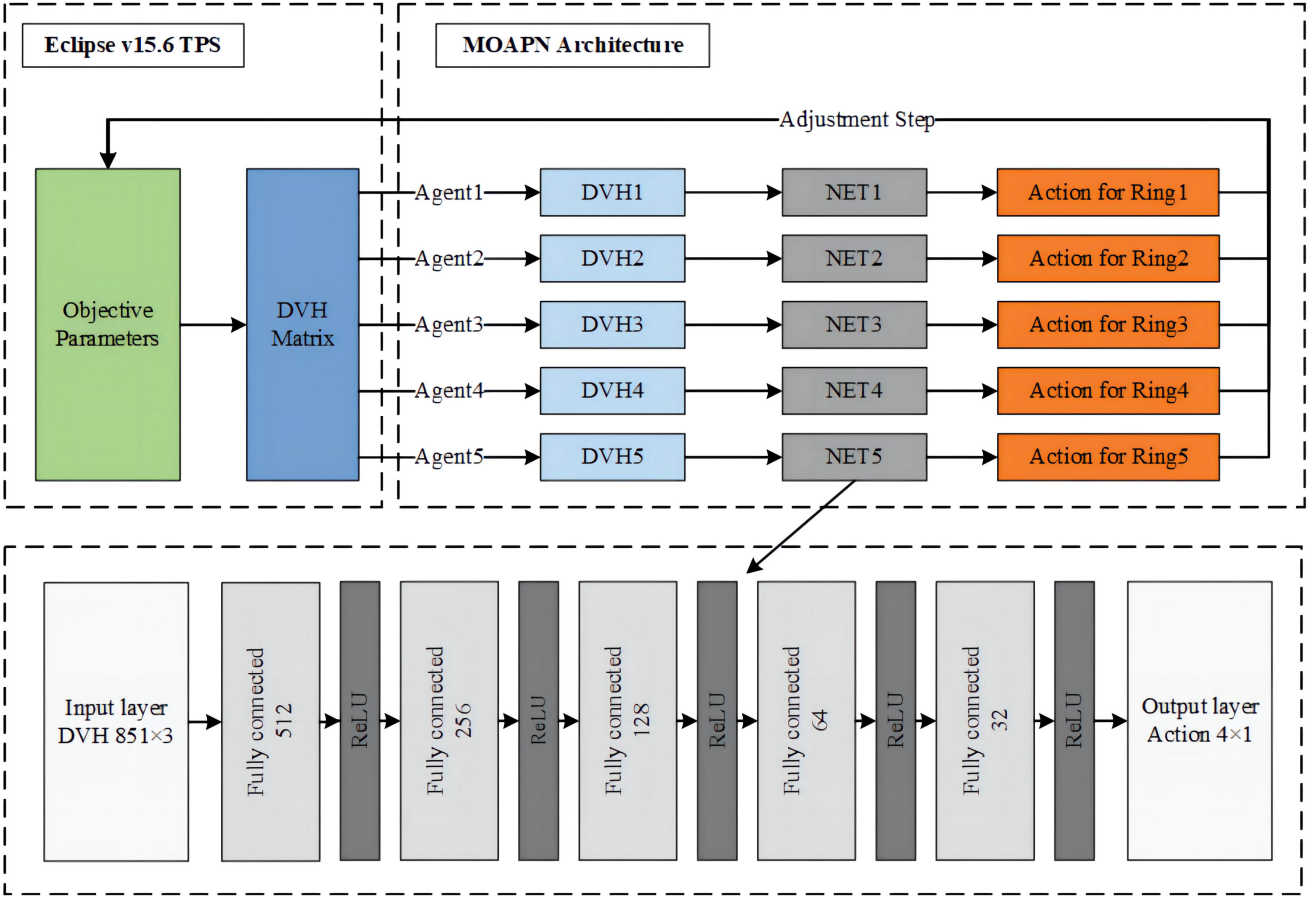
The network architecture of the MOAPN.

In the MOAPN, the state was defined as input data, which was the DVH matrix (850×12) of an optimized plan for each set of objectives, including PTV, all ring structures, lung, heart, spinal cord, esophagus, bronchus and chest wall. To shorten the training process and increase the robustness of network, we used the dose prediction module to obtain patient-specific initial optimization objectives. The MOAPN consisted of five similar DDQN networks controlled by five agents and with independent input data and memory buffers, as shown in **Figure 2**. During optimization, four input parameters were used for each optimization objective: an optimization weight factor, a 2D position goal on the DVH graph, and Boolean variable describing the direction of the constraint, as described in Section 2.3. In this model, the optimization weight factors of all structures were maintained at fixed values of 300 and 150 for the target objectives and other structures’ objectives, respectively. This indicates that more attention was paid to the target than other structures in planning optimization. The objective values of five ring structures obtained from the dose-volume goal on the DVH graph were adjusted to achieve desired dose gradient outside the target. Because the dose of OARs was affected by the adjustment of the ring dose, the dose constraints of all OARs were not adjusted in the optimization. When the ring dose reached the established requirements, the dose of OARs could meet the clinical requirements. Input data for each DDQN network were included in three columns corresponding to the DVHs of PTV, body and ring structure. In addition, we defined four possible adjustment actions for each objective: (a) 0-action: decrease the objective by 10%; (b) 1-action: decrease the objective by 3%; (c) 2-action: keep the objective unchanged; (d) 3-action: increase the objective by 5%. The network output was Q value matrix for all actions.

#### 2.5.3 Reward function

To evaluate the plan quality, we used a plan quality scoring system Eva(s). The Eva(s) scoring system was derived from the Plan IQ evaluation system (Sun Nuclear, Melbourne, FL), which included partial functions considered applicable to our study. The scoring system consisted of a set of criteria for evaluating plan quality based on target coverage, dose gradient and spring of OARs. Following the UK 2022 consensus (32) and RTOG 0915 report (22), the Eva(s) gave stricter criteria to achieve better plan quality. For the *D*_95%_ of PTV, the scoring criterion was defined as a piecewise linear function. No penalty occurred when the prescription dose was met. Once the actual dose was below the prescription dose, this criterion score rapidly decayed. Other scoring criteria were defined as once-linear functions to reduce the surrounding OARs dose as much as possible while maintaining the clinical prescription dose. The components of the Eva(s) are shown in **Table 1**.

**Table 1:** Plan quality metrics and assigned scores for assessing automatic plan quality.

Structure	Metric	Scoring Criterion
PTV	D_95%_ (Gy)	$$\text{score}=\{\begin{array}{c} 100,\;if\;D_{95\%}\geq 50\;\\ 100*\left(D_{95\%}-48.5\right)/\left(50-48.5\right)\;\;if\;D_{95\%}<50 \end{array}$$
PTV	CI	score=100*(CI−0.75)/(1−0.75)
PTV	GI	score=100*(GI−3.75)/(3−3.75)
Ring1	D_max_ (Gy)	score=100*(*D*_*r* *e* *f*_−*D*_*m* *a* *x*_)/500
Ring2	D_max_ (Gy)	score=100*(*D*_*r* *e* *f*_−*D*_*m* *a* *x*_)/500
Ring3	D_max_ (Gy)	score=100*(*D*_*r* *e* *f*_−*D*_*m* *a* *x*_)/500
Ring4	D_max_ (Gy)	score=100*(*D*_*r* *e* *f*_−*D*_*m* *a* *x*_)/500
Ring5	D_max_ (Gy)	score=100*(*D*_*r* *e* *f*_−*D*_*m* *a* *x*_)/500
Chest wall	D_max_ (Gy)	score=50*(50−*D*_*m* *a* *x*_)/(50−20)
Spinal cord	D_max_ (Gy)	score=50*(15−*D*_*m* *a* *x*_)/15
Heart	D_max_ (Gy)	score=50*(15−*D*_*m* *a* *x*_)/15
Lung (AS)	V_5_ (%)	score=50*(35−*V*_5_)/35
Bronchus	D_max_ (Gy)	score=50*(15−*D*_*m* *a* *x*_)/15
Esophagus	D_max_ (Gy)	score=50*(15−*D*_*m* *a* *x*_)/15

D_max_, maximum dose; D_ref_, reference dose; D_x%_, absolute dose received by x% of the volume; V_x_, relative volume receiving > x Gy absolute dose; CI, conformity index; CI, (TV_RI_/TV)×(TV_RI_/V_RI_). TV_RI_ is the target volume covered by the prescription dose, TV the target volume, V_RI_ the volume covered by the prescription dose; GI, gradient index, GI = V_50%_/V_p_, V_50%_ is the volume covered by the 50% of the prescription dose, V_p_ is the volume covered by the prescription dose; Lung (AS), lung (affected side).

With the scoring system Eva(s), the reward function in MOAPN can quantify the plan quality change caused by objectives adjustment. The reward function is defined as follows:


(8) $$\text{r}=\{\begin{array}{c} -200,\;\;\;if\;D_{95\%}\left(PTV\right)<95\%\times D_{p}\\ -50,\;\;\;if\;D_{actual}\left(ring^{i}\right)\leq D_{obj}\left(ring^{i}\right)\;and\;a_{t}\left(ring^{i}\right)=3\\ -20,\;\;\;if\;Eva\left(s_{t+1}\right)<Eva(s_{t})\\ 5,\;\;\;if\;Eva\left(s_{t+1}\right)=Eva\left(s_{t}\right)\\ 20,\;\;\;if\;Eva\left(s_{t+1}\right)>Eva\left(s_{t}\right) \end{array}$$

This reward function assigned a positive reward when the plan score increased at the next step. In contrast, a negative reward was given when the plan score decreased. We awarded a large penalty value to avoid adjusting objectives in the wrong direction. In addition, when the plan score was unchanged, we assigned a smaller positive reward to encourage MOAPN to maintain the current state to avoid non-convergence caused by endless exploration for a better state. In the training process, we allowed MOAPN to search for a better state by sacrificing a certain amount of target coverage. A decay factor was defined to penalize plan score when the target did not meet the prescription dose. When the target coverage was below the minimum limitation, we assigned a bigger negative reward and stopped the optimization.

#### 2.5.4 Prioritized experience replays

In the training process, the states *s_t_* and *s_t_*_+1_, the chosen action *a_t_*, and the reward *r_t_* were stored as a transition (*s_t_*, *a_t_*, *r_t_*, *s_t_*_+1_) in the experience replay pool. The temporal difference error (TD-error) which represented the difference in Q value between the evaluation-network and target-network was used to update network parameters in MOAPN. Based on the TD error, the Sum-Tree structure was introduced to give sampling priority to each transition stored in the experience replay pool (33), which was constantly updated for each transition during training. When sampling from the experience replay pool, samples with larger TD-error were easier to be sampled.

#### 2.5.5 Training strategy

The MOAPN was trained on 11 patient cases selected from the total study cohort. All training cases included the patient-specific initial optimization objectives by dose prediction module, as shown in **Table 2**. For each training case, five DDQN networks independently performed three processes: objectives adjustment, transition storage and sample, and network training. After all objectives were adjusted, an optimization process was performed. In each step, the ϵ greedy algorithm was used for MOAPN to select actions. Specifically, each DDQN network randomly selected its own action among all possible actions with a probability of ϵ. Otherwise, the optimal actions that achieved the highest output value were selected with a probability of 1 - ϵ. The probability ϵ was started at 0.95 and decayed at a rate of 0.95/episode along the training process, with a minimum of 0.1. Considering that there were five objectives to adjust, five independent experience replay pools each with maximum capacity of 4096 were generated to store transitions (*S_t_*, *a_t_*, *r_t_*, *S_t_*_+1_) based on the Sum-Tree structure. In each iteration, 20 training samples were selected to update the parameters *θ^eva^* of evaluation-network. The parameters *θ^tar^* of target-network were replaced with those of evaluation-network after every 80 steps. The processes of adjustment, optimization and training were repeated for each training case until either of the following termination criterion was met: (1) a maximum number of adjustment steps of 20 was reached; (2) the target coverage reached the minimum limitation, i.e. *D*_95%_ (*PTV*)< 95% ×*D_p_*

**Table 2:** The initial patient-specific objectives of two representative cases based on the 3D dose prediction module.

Structure	Objective type	Parameter a/Volume (%)	Patient 1		Patient 2	
			Dose (Gy)	Weight	Dose (Gy)	Weight
PTV	Point (lower)	Volume=100	52.0	300	52	300
PTV	Point (upper)	Volume=0	67.5	300	67.5	300
Ring1	gEUD (upper)	a = 40	51.4	150	52.2	150
Ring2	gEUD (upper)	a = 40	35.4	150	34.9	150
Ring3	gEUD (upper)	a = 40	23.4	150	21.1	150
Ring4	gEUD (upper)	a = 40	18.2	150	14.0	150
Ring5	gEUD (upper)	a = 40	15.2	150	8.5	150
Chest wall	gEUD (upper)	a = 40	41.1	150	37.1	150
Spinal cord	gEUD (upper)	a = 40	6.8	150	6.5	150
Esophagus	gEUD (upper)	a = 40	10.2	150	7.3	150
Heart	gEUD (upper)	a = 40	14.9	150	24.1	150
Lung(AS)	gEUD (upper)	a = 1	8.6	150	5.5	150
Bronchus	gEUD (upper)	a = 40	12.8	150	4.0	150

Point (lower), the type of optimization objective function is dose-volume objective (minimum constraint); Point (upper), the type of optimization objective function is dose-volume objective (maximum constraint); gEUD (upper), the type of optimization objective function is gEUD objective (maximum constraint); Lung (AS), lung (affected side).

All the computations were performed using Python on a Varian desktop workstation with 16 Intel Xeon Silver 4110 CPU processors, 32GB memory and 2 NVIDIA Quadro P5000 GPU cards. Based on the Python-ESAPI, the Pycharm software was used to interact with TPS and debug all scripts.

#### 2.5.6 Evaluation

MOAPN was evaluated on a total of 27 cases were selected from the study cohort. The prescription dose for all cases was 50 Gy/5 fractions. Based on the dose prediction module, the optimization for each case started with patient-specific initial optimization objectives. The trained MOAPN was used for objectives adjustment and optimization. The iteration was terminated if the target did not meet the prescription dose, or the maximum number of 25 adjustment steps was reached. Wilcoxon signed rank test was used to investigate the significance of the difference in the final plans versus the initial plans and the clinical plans, with a statistically significance threshold set at P value< 0.05.

## 3 Results

In this study, the MOAPN training for lung SBRT cases was successfully performed, and the training time was about 3 days. The automatic treatment plan for each tested case was generated by MOAPN. The completed planning optimization process took about 5-6 min.

**Figure 3:**
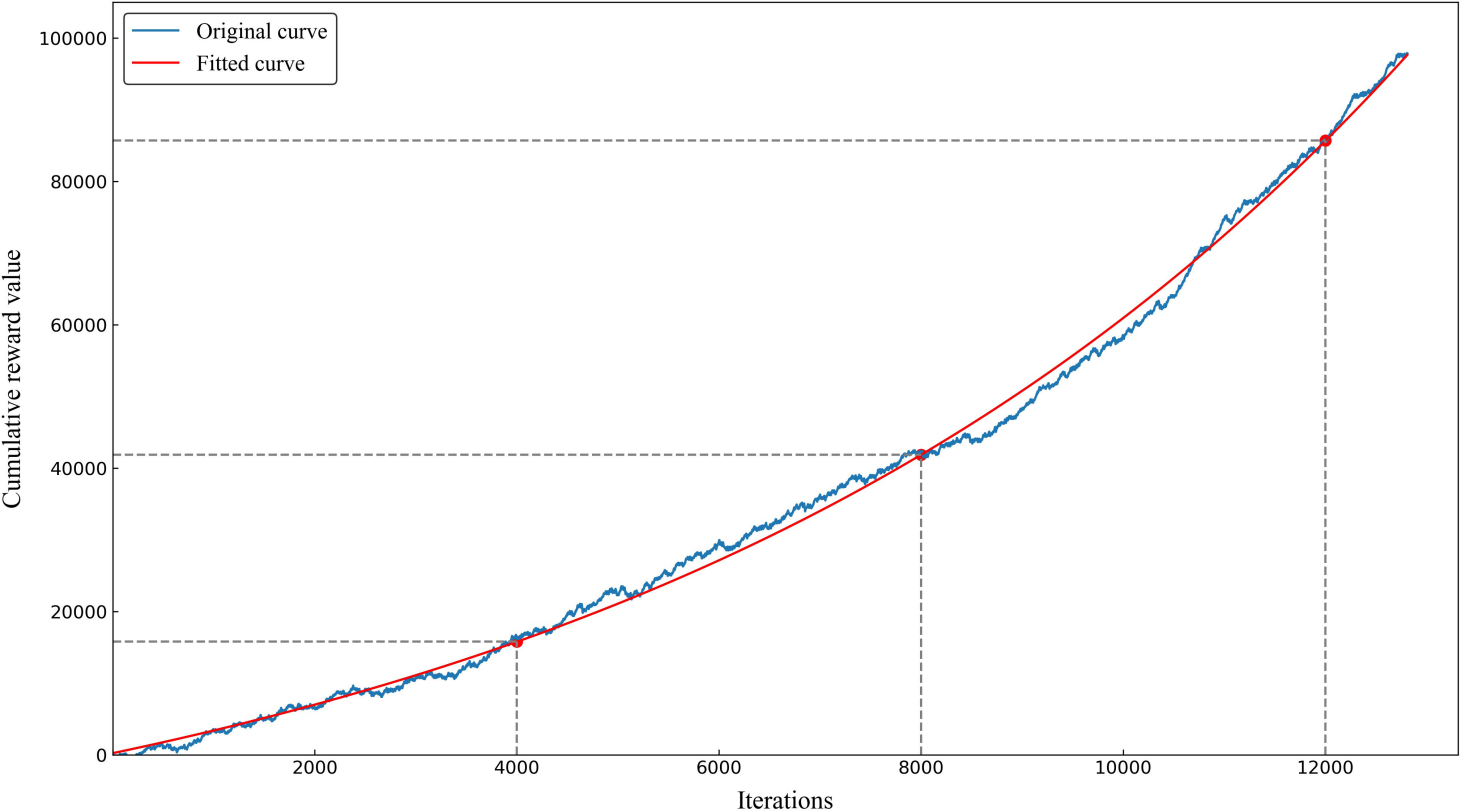
Correlation between the total training iterations and the cumulative reward value.

For all training cases, the relationship between the total training steps and the cumulative reward value is shown in **Figure 3**. The effectiveness of the proposed MOAPN framework was indicated by the increasing trend in rewards along the training steps. In the original curve, the cumulative reward value was found to fluctuated slightly. This phenomenon reflected that MOAPN used the trial-and-error approach to explore the optimal action-value policy. In the fitted curve, the cumulative reward per 4000 iterations increased by 65% and 67%, which indicated that MOAPN gradually learned objectives adjustment policy to improve the plan quality.

**Figure 4:**
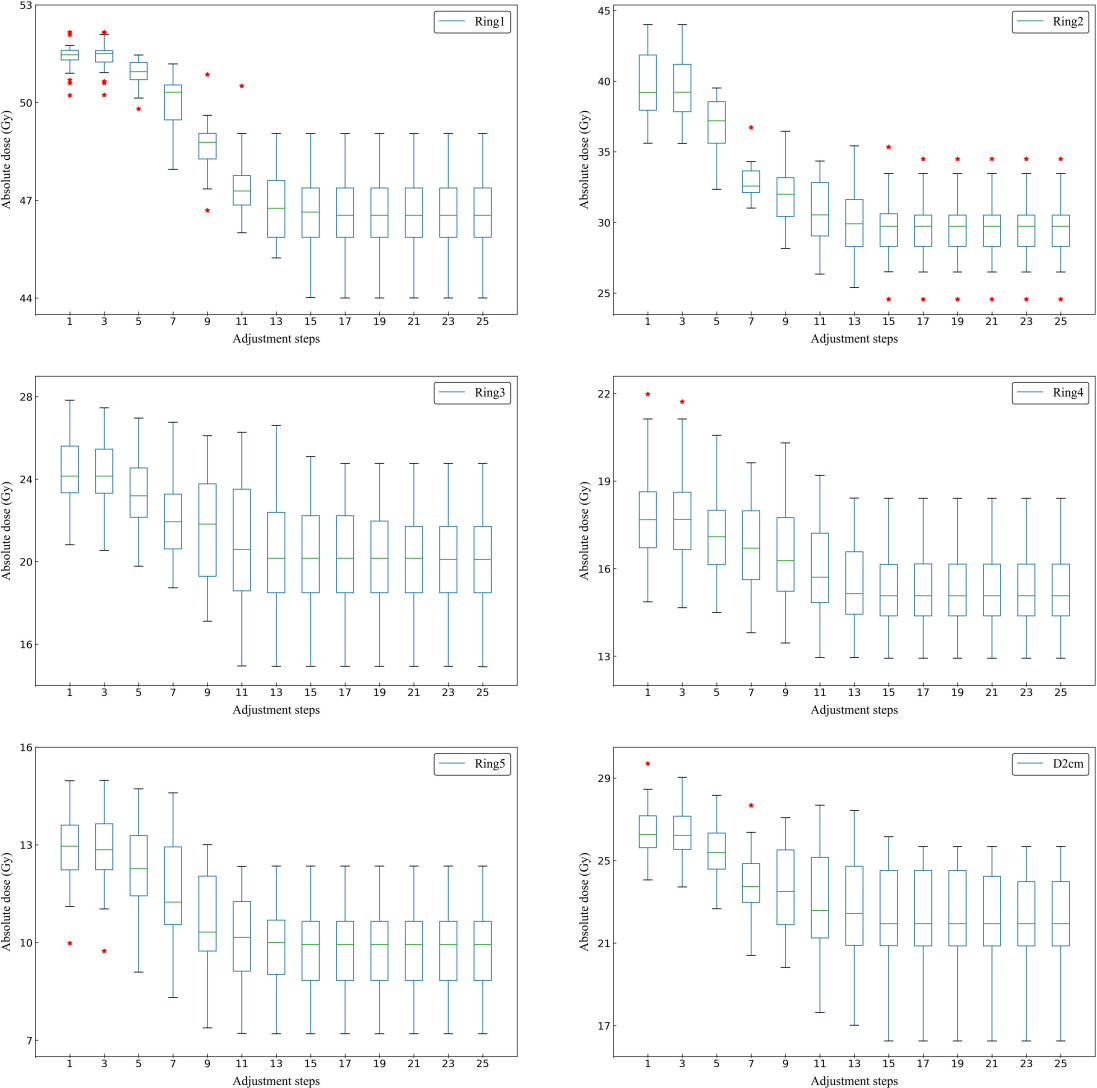
Box plots showing the relationship between the adjustment steps and the maximum dose of Ring1, Ring2, Ring3, Ring4, Ring5 and D2cm in all tested cases. The symbol * indicates some outliers present in the statistical values.

**Figure 4** shows the actual dose result of the ring structures of all tested cases in adjustment steps. The average number of adjustment steps for all tested cases was 21 ± 5.9. For all ring structures, the median, extremum and quartile of the average *D_max_* decreased significantly before 13 adjustment steps. After the 13th adjustment step, the median *D_max_* of all ring structures tended to converge. Therefore, a new case was able to complete optimization with about 13 adjustment steps by the trained MOAPN.

A summary of the quantitative comparison of MOAPN final plans, MOAPN initial plans and clinical plans for the OARs sparing is presented in **Table 3**. Compared with the MOAPN initial plans, the actual dose of the listed OARs in the MOAPN final plans decreased significantly except for the esophagus (p > 0.05). Among these, the average *D_max_* for the chest wall and the average for the affected side lung were reduced by 14.5% and 16.7%, respectively. However, the dose result of various OARs in the MOAPN final plans was similar to those in the clinical plans, e.g. spinal cord, heart, affected side lung, esophagus, bronchus and D2cm structure. Among these, the average *D_max_* for spinal cord, esophagus, bronchus and D2cm structure showed no significant difference (p > 0.05).

**Table 3:** Dosimetric parameters comparison between MOAPN final plans versus MOAPN initial plans and clinical plans (mean ± standard deviation).

Structure	Parameter	Final plans	Initial plans	P value	Clinical plans	P value
Chest wall	D_max_ (Gy)	37.02 ± 11.44	43.33 ± 9.27	<0.001	42.50 ± 9.44	<0.001
Spinal cord	D_max_ (Gy)	8.05 ± 3.94	9.11 ± 5.43	0.044	8.06 ± 5.46	0.885
Heart	D_max_ (Gy)	9.24 ± 6.97	9.70 ± 6.89	0.01	9.91 ± 8.01	0.031
Lung (AS)	_5_ (%)	24.51 ± 9.31	29.45 ± 9.08	<0.001	25.26 ± 7.87	0.037
Esophagus	D_max_ (Gy)	8.21 ± 3.86	8.37 ± 3.78	0.683	7.26 ± 3.77	0.064
Bronchus	D_max_ (Gy)	10.70 ± 5.64	11.60 ± 5.61	0.017	10.84 ± 5.59	0.904
D2cm_PTV	D_max_ (Gy)	22.14 ± 2.29	26.47 ± 1.23	<0.001	22.25 ± 2.18	0.597

Lung (AS), lung (affected side); D_max_, maximum dose; V_x_, relative volume receiving > x Gy absolute dose; P value, The P values in the first and second column represent the statistical results of the final plans versus the initial plans and the clinical plan, respectively.

**Figure 5:**
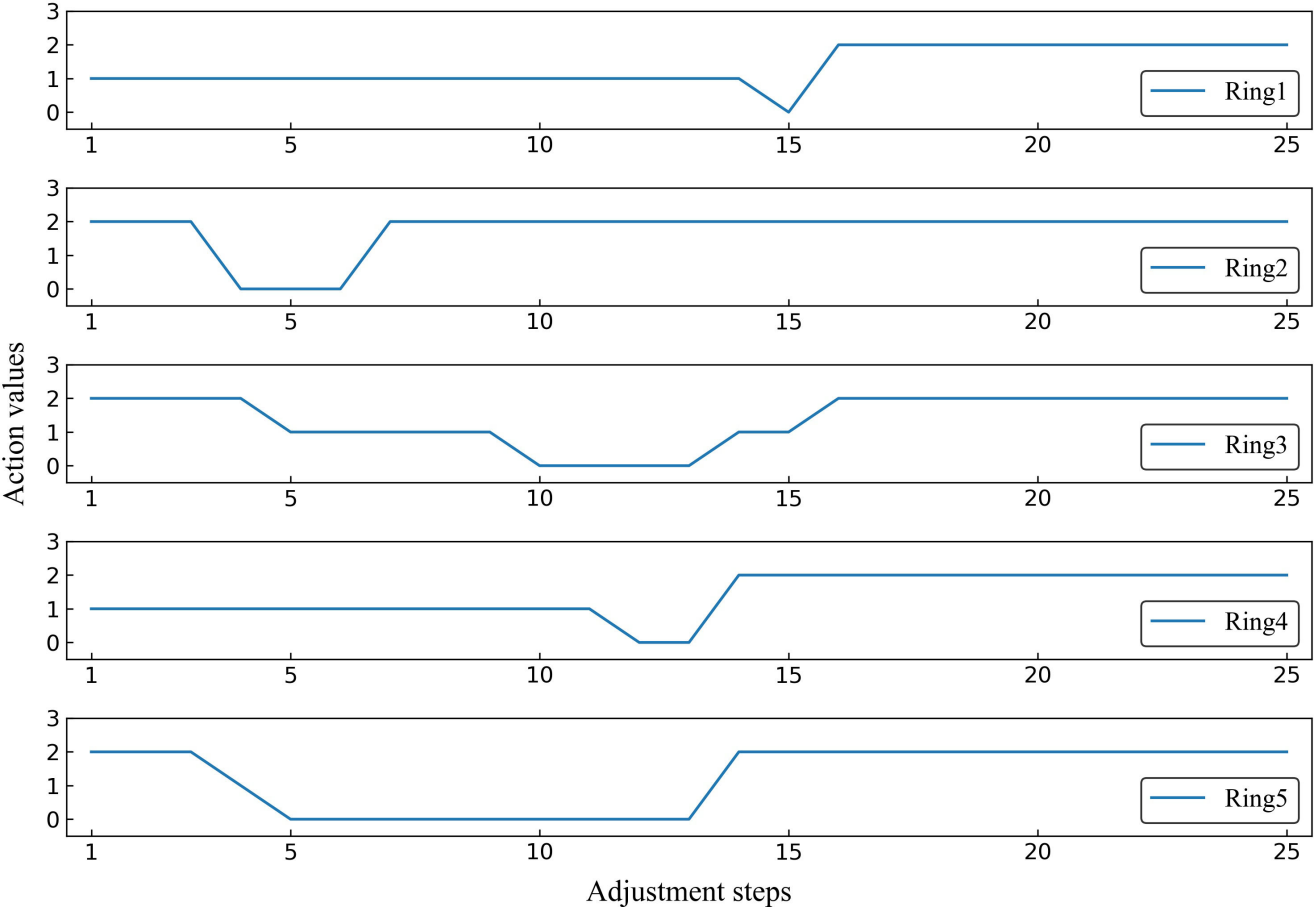
The selected action values of Ring1, Ring2, Ring3, Ring4 and Ring5 in the adjustment steps for one representative case.

**Figure 6:**
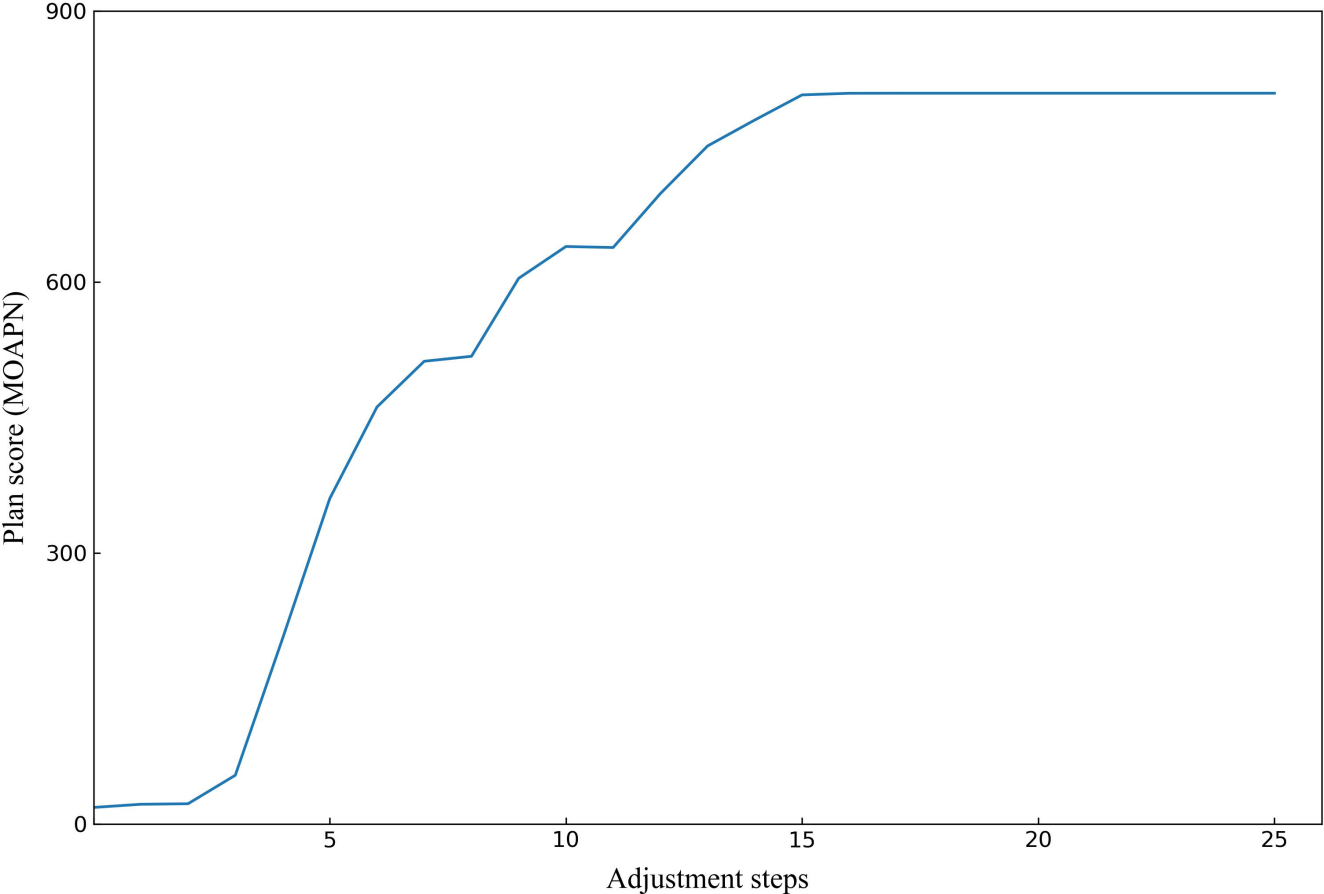
The obtained plan score of one representative case in the MOAPN adjustment steps.

To better demonstrate the effectiveness of MOAPN, the result of one representative case is shown in **Figures 5**, **6**. In the first 10 adjustment steps, the 0-action and 1-action were frequently selected for five ring structures to decrease the corresponding objectives significantly or slightly, respectively. After 15 adjustment steps, the 2-action was selected for each ring structure to keep the objectives unchanged. In addition, the Ring2 selected the 2-action 23 times in overall adjustment steps, indicating that the result of dose prediction module had basically reached the desired objective of MOAPN. The advantages of MOAPN can also be observed visually through the planning score in overall adjustment steps, as shown in the **Figure 6**. The average plan score increased from 18.3 to 809.1 and converged after 15 adjustment steps.

**Figure 7:**
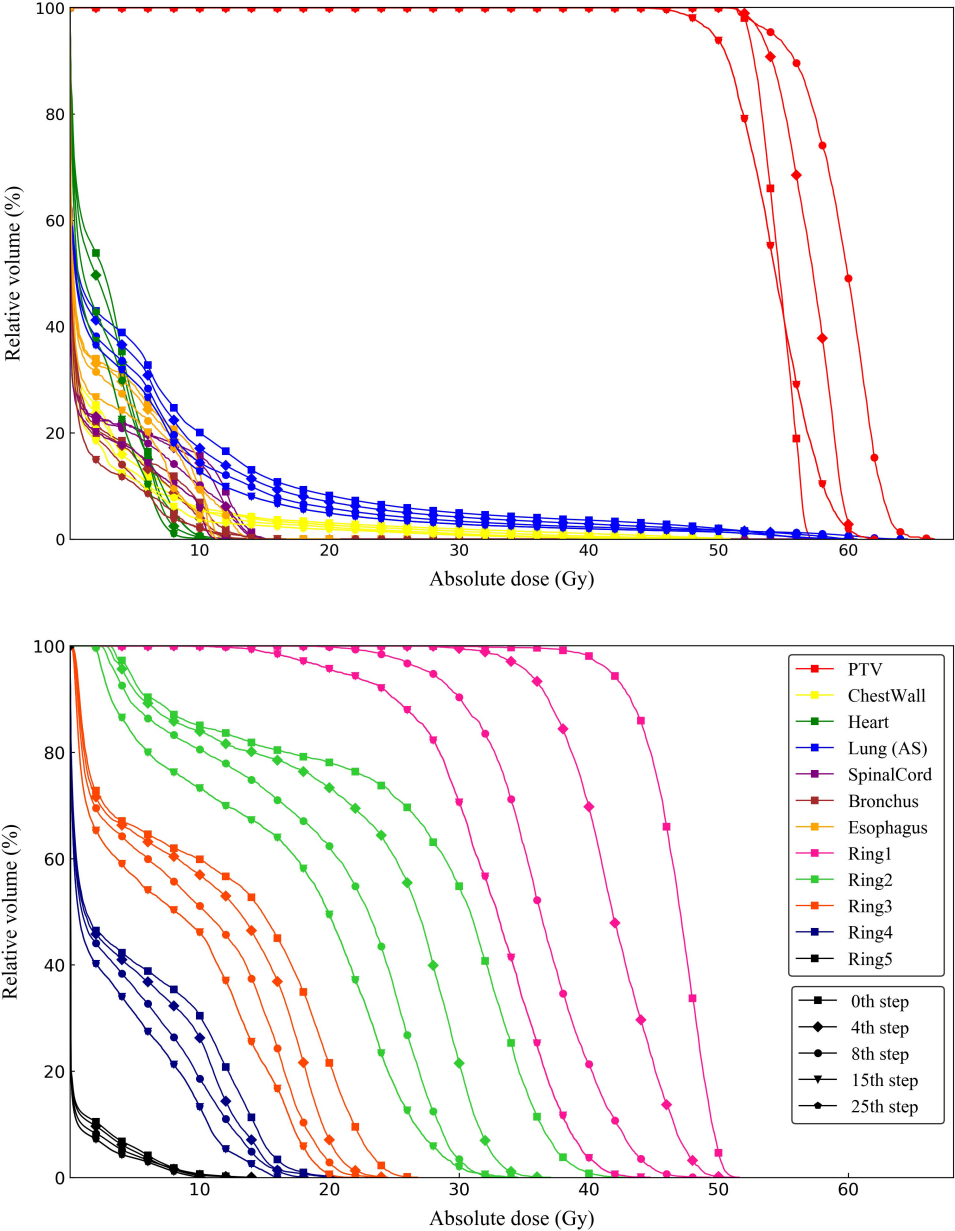
DVH curves of one representative case between 0th, 4th, 8th, 15th and 25th adjustment steps compared from ring structures and OARs.

**Figure 8:**
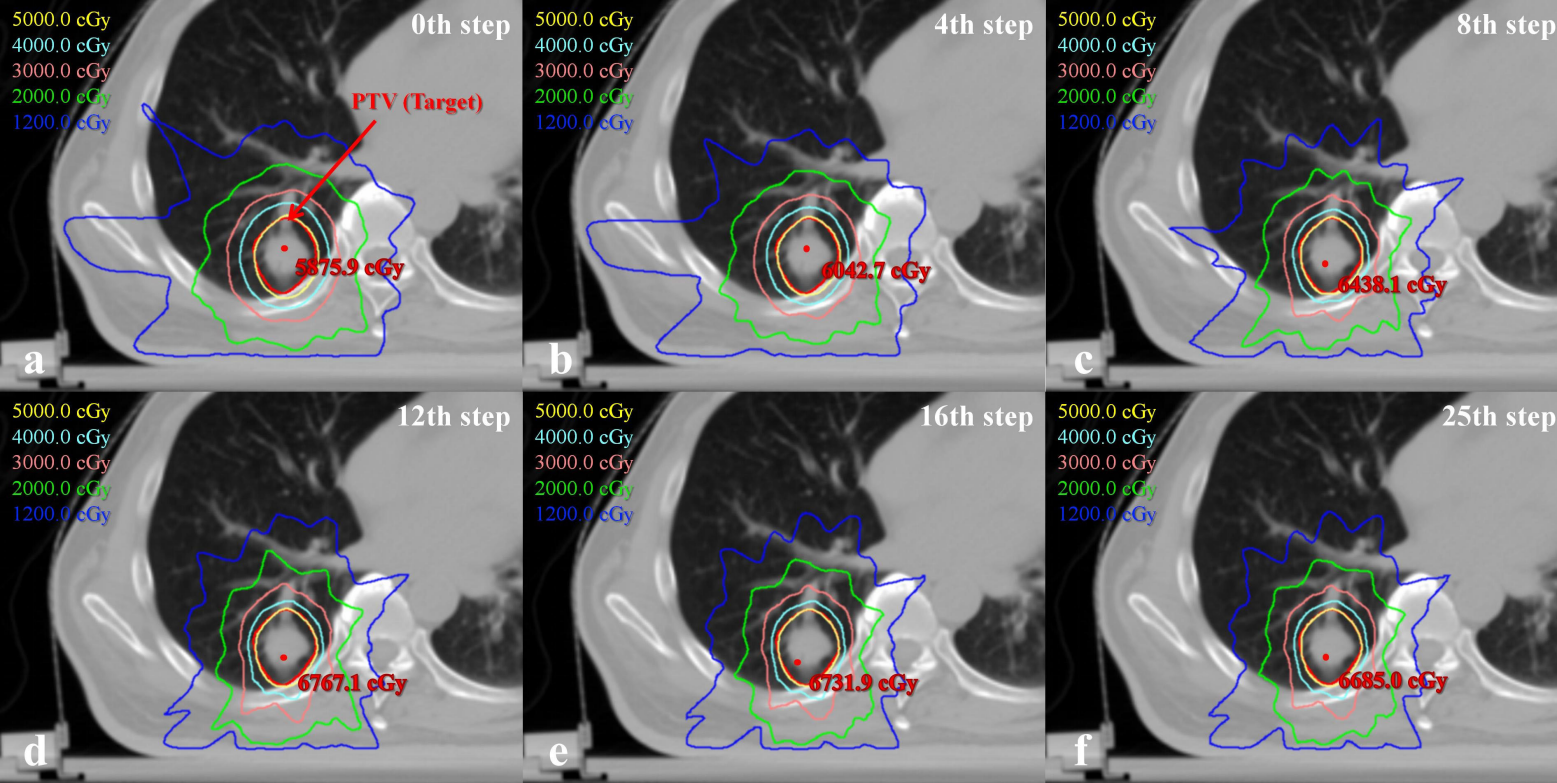
Comparison of the transverse isodose distributions between 0th, 4th, 8th, 12th, 16th and 25th adjustment steps for one representative case.

To better understand plan quality changes, the DVH curves of all structures for the representative case between the 0th, 4th, 8th, 15th and 25th adjustment steps are compared in **Figure 7**. Obviously, the significant improvement in the DVH curves can be observed in five ring structures. Affected by adjusting the ring structure objectives, the dose of OAR decreased to varying degrees. In addition, we found that the 15th DVH curve and 25th DVH curve of various structures basically coincided, indicating that the representative case had converged and tended to the optimal plan at the 15th adjustment step. The comparison of the isodose distribution for the representative case between the 0th, 4th, 8th, 12th, 16th and 25th adjustment steps is shown in **Figure 8**. Before the 12th adjustment step, it can be visually observed that the isodose lines tightened continuously and the value of the maximum dose point of the transverse slice kept increasing. It should be noted that the comparison results of isodose distribution in other tested cases were also similar.

## 4 Discussion

Based on ESAPI, this study developed an integrated solution for automatic treatment planning. First, a 3D dose prediction module was built to obtain patient-specific initial optimization objectives. With the help of a multi-agent DRL scheme, the MOAPN was developed and trained to operate an optimization engine to generate high-quality plans. The clinical feasibility of automatic optimization of radiotherapy plans based on the Eclipse TPS was demonstrated. The MOAPN generated an action-value policy similar to the physician’s iterative operation during the planning optimization. With this integrated solution, a more efficient overall workflow of treatment planning was achieved.

Numerous studies have reported RL-based automatic planning for different tumor sites. Hrinivich et al. proposed a RL VMAT algorithm in a in-house developed TPS, which can learn a machine control policy using previous cases geometry (17). This policy was used for new cases to rapidly optimize treatment plans and generate sequences of deliverable machine parameters without adjusting optimization objectives. Shen et al. constructed a hierarchical virtual treatment planner network consisting of structure-net, parameter-net and action-net (34). This network selected the structure and parameter to adjust and determined the specific adjustment sequence, similar to the behavior of a planner. Compared with in-house automatic planning strategy, our approach used the commercial TPS optimization engine and algorithms to adjust the optimization parameters and generate dose distribution. To our knowledge, this is the first RL implementation for automatic IMRT planning *via* ESAPI. The main limitation is that the Eclipse’s optimization algorithm is treated as a black box in iteration process. Hrinivich’s approach used the uniform objective map, which cannot optimally reflect the variation of cases. With the objective values met, further improvement was not needed. Based on dose prediction result, our approach further searched patient-specific optimization parameters, which helped achieve a better plan quality. In addition, Shen’s hierarchical DRL network shows a good application in parameter decision-making process when the number of optimization parameters is sufficiently large. We aim to further enhance the hierarchical framework of MOAPN to improve its application to complex clinical situations.

The anatomical geometry of the tumor and body varies from case to case, and this difference can exert various effects on the final dose distribution. Unreasonable objectives will enlarge the parameter space explored, resulting in a time-consuming trial-and-error process in optimization. Therefore, it is necessary to obtain a set of patient-specific optimization parameters for each patient case (5). In our study, the dose prediction module was summarized into two steps: predicting 3D dose distribution by training and generating the two-dimensional dose-volume parameters. However, a major limitation of this KBP approach is that the results are strongly dependent on the training database used. Higher performance of the dose prediction model can be achieved with a sufficiently large, high-quality planning database. If the ground truth doses are suboptimal, the predicted doses will also be suboptimal. Moreover, the dose prediction model need to consider the unique clinical characteristics of each patient case, including PTV size, which shows significant variability and spatial complexity of neighboring anatomy. However, the KBP approach has region-specific features, as it is typically used with independent databases and models in different treatment centers to improve the accuracy of the prediction result. This limits the universal applicability of effective planning models in different regions. In our study, the integrated solution combines the respective advantages of the dose prediction module and DRL method. This precludes the need for optimal prediction results. The 3D dose prediction module is utilized to obtain the initial patient-specific initial optimization objectives. In addition, with this module, uniform optimization objectives are not necessary, which minimizes the parameter search space and optimizes computational resources. When the predicted results are ideal, MOAPN can complete the planning optimization process in a few iterations. Even with an inaccurate predicted dose, the action-value policy formed by MOAPN has the ability to adjust objectives and gradually generate a satisfactory plan. Therefore, this integrated solution has a wide application potential in different regions and treatment centers. In addition, the patient-specific initial optimization objectives can not only better reflect the variation of cases, but also contribute to faster convergence of MOAPN in training process and improve the overall work efficiency.

This study focused on a problem of lung SBRT automatic treatment planning for a less complex case with moderate number of OARs. Five dose-limiting ring structures were generated using a prewritten script and used to control the dose gradient outside the target to an acceptable level without adjusting the dose constraints of all OARs. In **Table 3**, the experimental result shows that when the objectives of ring structures are adjusted, the dose of OARs can be controlled to clinically acceptable levels. Compared with the single-agent model, the MOAPN can provide independent training process for each objective and simultaneously adjust multiple objectives based on the current state. Thus, this method could improve the work efficiency and reduce the variation of plan quality in clinical application. As this method is established using the ESAPI module, it can be readily integrated into any Eclipse TPS (v. ≥ 15.6) as a plug-in application. Script-based automatic approaches have been proven to have great potential for reducing workloads in clinical radiotherapy (35). In addition, it is very flexible and can be modified for various prescriptions, tumor sites (e.g. rectum and head-and-neck), and OARs. In a follow-up study, we aim to develop an automatic planning assistance module for TPS using ESAPI. Planners can directly use the automatic planning design program by inputting the relevant parameters in the prompt box, without fully understanding the code architecture of this method, providing more convenience for clinical work.

However, the current study still has several limitations. First, to demonstrate the feasibility of the proposed method, we constructed a simple plan quality scoring system and reward function. As these may not fully reflect the clinical criteria for plan quality evaluation, it is necessary to add more clinical criteria to the reward function. In addition, this study only illustrated the feasibility of the MOAPN approach in only a few cases. A large number of cases are required to find and seal loopholes in its clinical implementation and improve robustness in the handing of clinical cases. The quality of automatic planning generated by MOAPN may also not achieve clinical acceptability by physicians. Second, the MOAPN only had four action options for adjusting the objectives, which may affect the ability of adjustment to some degree. To provide diversified adjustment steps, we aim to improve MOAPN in the future to implement the continuous action control based on the current state (36). Third, the input feature of MOAPN only was based on DVH matrix since DVH is a concise representation of plan quality. However, it is well-known that DVH cannot capture spatial location information such as hot/cold spots, which is critical to physician decisions. We expect that the 3D dose information will be used to handle more complicated tasks in the future. Fourth, this study is a new attempt at automatic planning. However, the current application scope of the automatic plans is limited to peripheral NSCLC cases. Based on the original model, we have introduced more tumor sites data for training to obtain a more general model in the future. Nevertheless, it must be acknowledged that these improvements inevitably lead to a larger MOAPN architecture and higher computational burden, such as when training with 3D dose information, adding more adjustable objectives, and handling more complex plans. We need to upgrade hardware devices and software to improve the efficiency of algorithm execution. Although our method shows good performance and great potential in the field of automatic planning, it does not completely replace manual intervention, especially for some complex cases. If the planners are not satisfied with the results of the automatic planning, the obtained plan can be an intermediate step for further manual intervention, which can accelerate the trial-and-error process.

## 5 Conclusion

With the help of ESAPI, we proposed an effective and efficient integrated solution for automatic treatment planning. It first includes the dose prediction module for obtaining patient-specific initial optimization objectives. We demonstrated that the trained MOAPN can mimic the operations of the physicians during optimization and adjust multiple objectives to obtain a high-quality plan in Eclipse TPS. The quality of automatic plans created by MOAPN shows progressive improvement during the adjustment step and is close to that of the clinical plan. Moreover, this integrated solution contributes to improving the efficiency of the overall planning workflow and reducing the variation of plan quality in clinical practice. In conclusion, the proposed integrated solution is a promising practical and effective approach for automatic planning in commercial TPS.

## Data availability statement

The original contributions presented in the study are included in the article/**Supplementary Material**, further inquiries can be directed to the corresponding author/s.

## Author contributions

HW is the first authors of this paper. HW contributed to every part of the whole research, including collecting clinical data sets, designing methodology, writing script codes and writing the manuscript et al. For the other authors, XB was responsible for the dose prediction module. YW and YL were responsible for providing clinical assistance and manual planning. HW and XB were responsible for reviewing data analysis result. XB and BW contributed to reviewing the manuscript. HW was responsible for the revision and improvement of the manuscript.

## Supplementary material

Date sheet 1 In this document, the dosimetric parameters for all tested cases are recorded between the MOAPN final plans, the clinical plans, and the MOAPN initial plans.

Date sheet 2 In this file is recorded that the actual dose of six ring structures (Ring1, Ring2, Ring3, Ring4, Ring5, D2cm) changes during the overall adjustment steps..
